# Structure of monkeypox virus poxin: implications for drug design

**DOI:** 10.1007/s00705-023-05824-4

**Published:** 2023-06-28

**Authors:** Vojtech Duchoslav, Evzen Boura

**Affiliations:** Institute of Organic Chemistry and Biochemistry, Academy of Sciences of the Czech Republic, v.v.i, Flemingovo nám. 2, 166 10 Prague 6, Czech Republic

## Abstract

Monkeypox, or mpox, is a disease that has recently resurfaced and spread across the globe. Despite the availability of an FDA-approved vaccine (JYNNEOS) and an effective drug (tecovirimat), concerns remain over the possible recurrence of a viral pandemic. Like any other virus, mpox virus must overcome the immune system to replicate. Viruses have evolved various strategies to overcome both innate and adaptive immunity. Poxviruses possess an unusual nuclease, poxin, which cleaves 2'-3'-cGAMP, a cyclic dinucleotide, which is an important second messenger in the cGAS-STING signaling pathway. Here, we present the crystal structure of mpox poxin. The structure reveals a conserved, predominantly β-sheet fold and highlights the high conservation of the cGAMP binding site and of the catalytic residues His17, Tyr138, and Lys142. This research suggests that poxin inhibitors could be effective against multiple poxviruses.

**Supplementary Information:**

The online version contains supplementary material available at 10.1007/s00705-023-05824-4.

Mpox, previously known as monkeypox, is a disease that has recently re-emerged [1]. It was previously endemic to central Africa, where rodents and non-human primates might serve as the natural reservoir of mpox virus and transmit it to humans. However, it has recently spread across the globe. Originally, it was reported to have a high mortality rate of about 3-6% [2], but that seems to have been an overestimate, at least for the currently dominant strains. An FDA-approved vaccine (JYNNEOS) is available [3], and at least one FDA-approved effective drug (tecovirimat) is also available [4]. Nonetheless, this virus has raised great concern about a possible recurrence of a viral pandemic and all the unpopular anti-pandemic measures that may be necessary. Considering the potential emergence of drug/vaccine-resistant strains and market shortages, it is prudent to ensure the availability of multiple drugs and vaccines.

Mpox virus (MPXV), like any other virus, must overcome the immune system to successfully replicate [5]. Innate immunity is of foremost importance in the early stages of a viral disease before adaptive immunity can intervene. Viruses have evolved many strategies to overcome innate and adaptive immunity [6]. The HIV negative factor (Nef) protein downregulates the CD4 and major histocompatibility complex I (MHC-I) proteins by hijacking the endocytic adaptor protein complexes AP-1 and AP-2 [7]. Coronaviruses such as SARS-CoV-2 and flaviviruses such as Zika virus and dengue virus have an RNA cap at the 5' end of their RNA that is chemically indistinguishable from the human RNA cap, and this prevents the innate immune system from detecting viral RNA in the cytoplasm [8, 9]. In fact, RNA capping was discovered to mediate effective translation of viral RNA in the case of vaccinia virus (family *Poxviridae*) already in the late 1970s [10]. Furthermore, like coronaviruses, poxviruses also possess RNA nucleases to prevent the accumulation of double-stranded RNA (dsRNA) in the cytoplasm, which would otherwise trigger an innate immune response [11]. However, poxviruses have developed additional strategies to evade the immune system, as comprehensively reviewed by Yu et al. [12].

Interestingly, poxviruses possess a nuclease that is rather unusual and was named after them – poxin [13, 14]. Its substrate is 2',3'-cyclic guanosine monophosphate-adenosine monophosphate (referred to as cGAMP), a cyclic dinucleotide that consists of AMP and GMP units cyclized via 3',5' and 2',5' linkages (Fig. 1). Poxins cleave the 3'-5' bond of cGAMP, effectively removing it from the cytoplasm [13, 15]. This is important for the survival of the virus because cGAMP is a second messenger that is produced by cGAMP synthase, an enzyme localized in the cytoplasm that is activated by the presence of DNA and is part of the defense against DNA viruses that replicate in the cytoplasm [16]. Upon activation of cGAMP synthase, cGAMP is produced and binds to STING (stimulator of interferon genes), inducing a conformational change and activating STING-dependent signaling. This process is often referred to as the cGAS-STING (cyclic GMP-AMP synthase - STING) signaling pathway, and poxins efficiently intercept it [17].

**Fig. 1:**
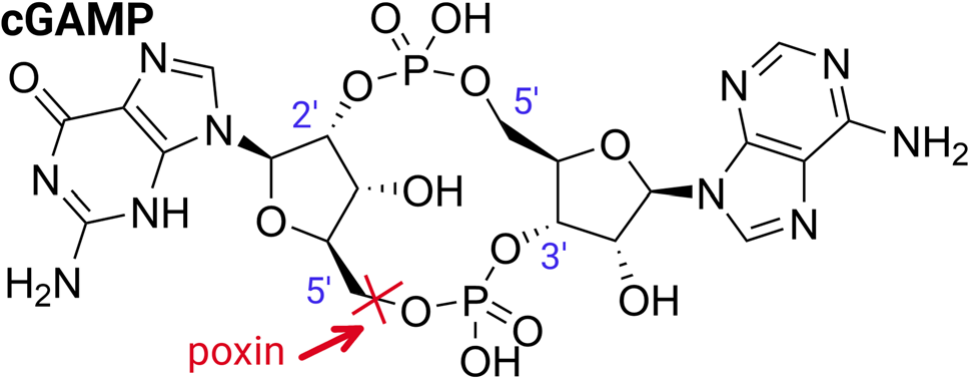
Chemical structure of cGAMP. The 2', 3', and 5' position of the ribose are highlighted in blue. The poxin cleavage site is shown in red.

In this study, we successfully determined the crystal structure of the poxin protein from mpox virus. We have identified a conserved hotspot that serves as the binding site for cGAMP and is a potential target for inhibitor design. Novel strategies based on inhibition of poxin, can be developed to combat mpox virus infections.

Certain poxviruses, including mpox virus, express the poxin protein as a fusion with a C-terminal schlafen domain [13]. However, the specific function of this domain remains unknown. Therefore, we aimed to solve the crystal structure of mpox poxin domain only. We prepared the recombinant mpox poxin using our usual protocols for viral enzymes [18, 19] as detailed in the supplementary information. After some optimization, we obtained crystals that belonged to the monoclinic P2_1_ spacegroup and diffracted to 1.7Å resolution. The structure was solved by molecular replacement using the vaccinia virus (VACV) poxin as a search model [13], and it was refined to good R-factors (R_work_ = 21.36%, R_free_ = 22.84%) and good geometry (Table 1).

**Table 1:** Statistics of crystallographic data and refinement

Crystal	Mpox poxin
PDB accession code	8C9K
Data collection and processing	
Space group	P2_1_
Cell dimensions	
a, b, c (Å)	54.43, 94.13, 94.67
α, β, γ (°)	90, 105.26, 90
Resolution range (Å)	41.09-1.72 (1.782-1.72)
No. of unique reflections	200402 (9723)
Completeness (%)	99.36 (99.72)
Multiplicity	3.4 (3.5)
Mean I/σ(I)	8.71 (0.84)
R-merge	0.1353 (2.239)
R-meas	0.1466 (2.418)
CC_1/2_ (%)	0.998 (0.462)
CC* (%)	0.999 (0.795)
Structure solution and refinement	
R-work (%)	21.36 (38.32)
R-free (%)	22.84 (41.23)
CC-work (%)	96.5 (68.6)
CC-free (%)	94.3 (42.5)
Ramachandran favored/outliers (%)	96.22/0
R.m.s.d.	
Bonds (Å)	0.003
Angles (°)	0.72

The structure revealed a predominantly β-sheet fold that could be divided into an N-terminal protease-like domain (NTD) and a C-terminal domain (CTD) (Fig. 2), similar to those found in the poxins of VACV [13] and baculoviruses [15]. The NTD is composed of twelve β-strands that form five antiparallel β-sheets, each composed of two or three β-strands (β-1&2&3, β-9&10, β-4&5, β-8&11, β-6&7&12). The CTD contains the only α-helix (α1) and two β-sheets, one consisting of two and the other of four β-strands.

**Fig. 2:**
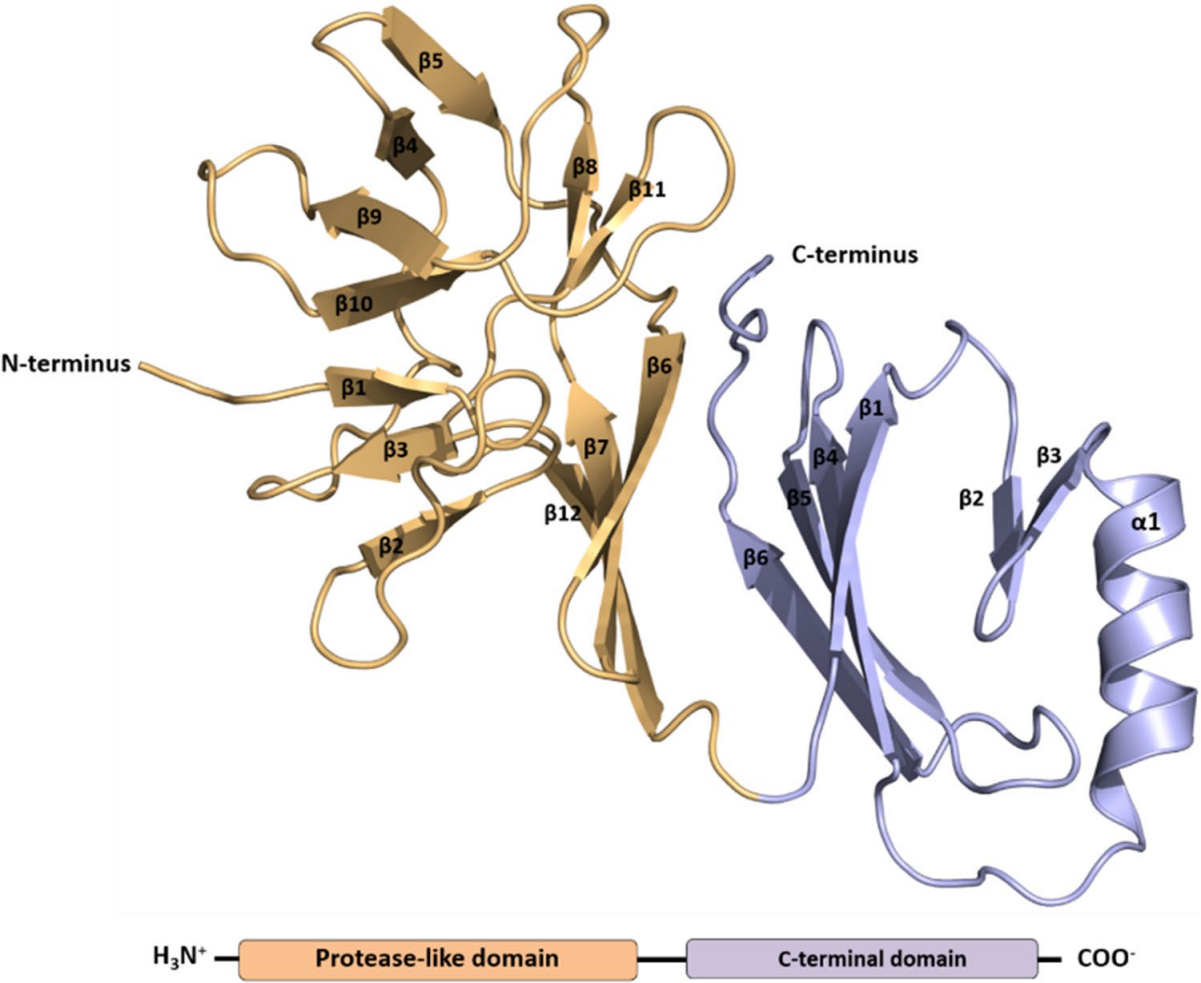
Structure of the mpox poxin. The mpox poxin can be divided in two domains. The first is the N-terminal or protease-like domain (depicted in gold), which comprises twelve β-strands that come together to form five β-sheets. The second is the C-terminal domain (depicted in cyan), which is composed of a single α-helix and six β-strands that form two β-sheets.

However, to form an active enzyme, poxin needs to form a dimer [13, 15], which we also observed in our crystal structure (Fig. 3). The dimer is held together by a network of hydrogen bonds that form between antiparallel β-strands of different subunits. Two novel β-sheets are formed by hydrogen bonding between β-strands from different poxin monomers (Fig. 3, right panels).

**Fig. 3:**
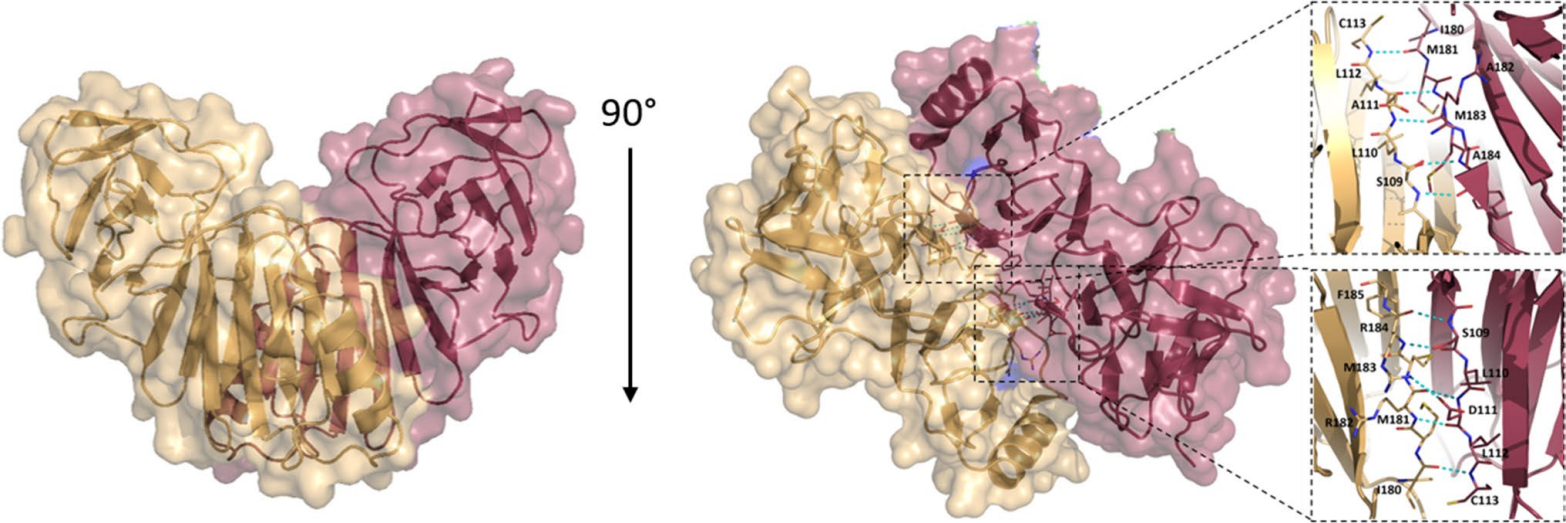
The poxin dimer. The active mpox poxin homodimer complex, shown in a surface representation, forms a heart-like shape. Poxin dimerization occurs through hydrogen bonds between antiparallel β-strands in both subunits.

The mpox poxin shares 91% identity with the VACV poxin (Supplementary Fig. S1). We analyzed the conservation of the catalytic residues. The cGAMP binding site is localized at the dimer interface, and His17 from one monomer and Tyr138 with Lys142 from the other were identified as the residues responsible for poxin-catalyzed cleavage of cGAMP, as in the case of the VACV poxin [13]. We superimposed the structures of the mpox and VACV poxins, revealing that these residues are conserved and in the same conformation in both of these proteins (Fig. 4).

**Fig. 4:**
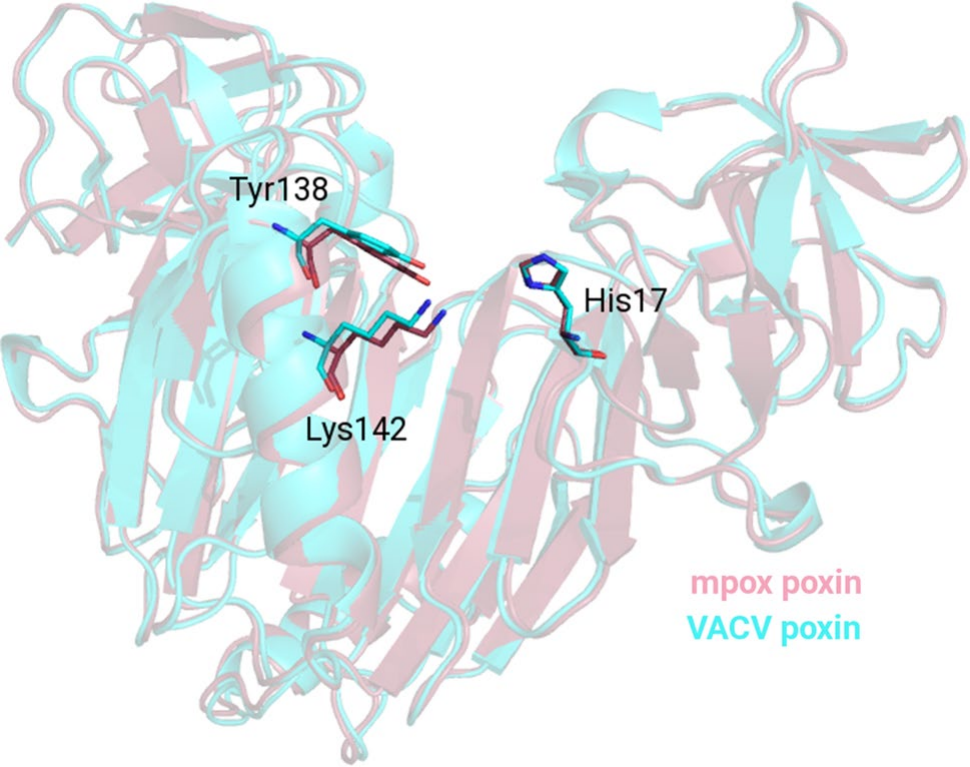
Conservation of the key catalytic residues in the mpox and vaccinia virus poxins

These findings indicate that poxin is a promising target for the development of drugs that can effectively combat multiple members of the family *Poxviridae*. However, the effectiveness of using poxin as an antiviral target requires the development of potent inhibitors to confirm its potential. Tecovirimat, the only FDA-approved drug against mpox, is an inhibitor of the envelope protein p37 [4, 20, 21]. Traditionally, most antiviral drugs target enzymes such as the polymerase, protease, or integrase. However, recently, especially during the COVID-19 pandemic, inhibitors of other enzymes such as RNA-methyltransferases have been reported by us and others [8, 22–25], and we also reported inhibitors of the mpox methyltransferase VP39 [26]. Interestingly, inhibitors of capsid proteins have also reached the market, most notably, Gilead Science's HIV capsid inhibitor lenacapavir [27, 28]. The natural ligand of poxin is the cyclic dinucleotide cGAMP, which normally activates STING. Recently, many cGAMP analogs have been prepared, and the medicinal chemistry of these compounds is now well understood [29–32]. Some of these have been shown to be resistant to cleavage by poxins [33]. We speculate that the use of these compounds will help to determine the exact role of poxins in the life cycle of poxviruses and establish the suitability of poxins as drug targets.

## Supplementary Information

Below is the link to the electronic supplementary material.

Supplementary file1 (PDF 841 KB)

## Data Availability

The data were deposited in the PDB database and are available under the PDB accession code 8C9K.
