# Supplementary material for: Structure of monkeypox virus poxin: implications for drug design
Source: Arch Virol. 2023 Jun 28;168(7):192. doi: 10.1007/s00705-023-05824-4 (PMC10307694; doi:10.1007/s00705-023-05824-4)
Supplement: Supplementary file 1 — Supplementary file1 (PDF 841 KB) [file 705_2023_5824_MOESM1_ESM.pdf]

Structure of monkeypox virus poxin: implications for drug design

Vojtech Duchoslav[a], Evzen Boura[a,*]

[a]Institute of Organic Chemistry and Biochemistry, Academy of Sciences of the Czech Republic, v.v.i, Flemingovo nám. 2, 166 10 Prague 6, Czech Republic.

[*] correspondence to boura@uochb.cas.cz

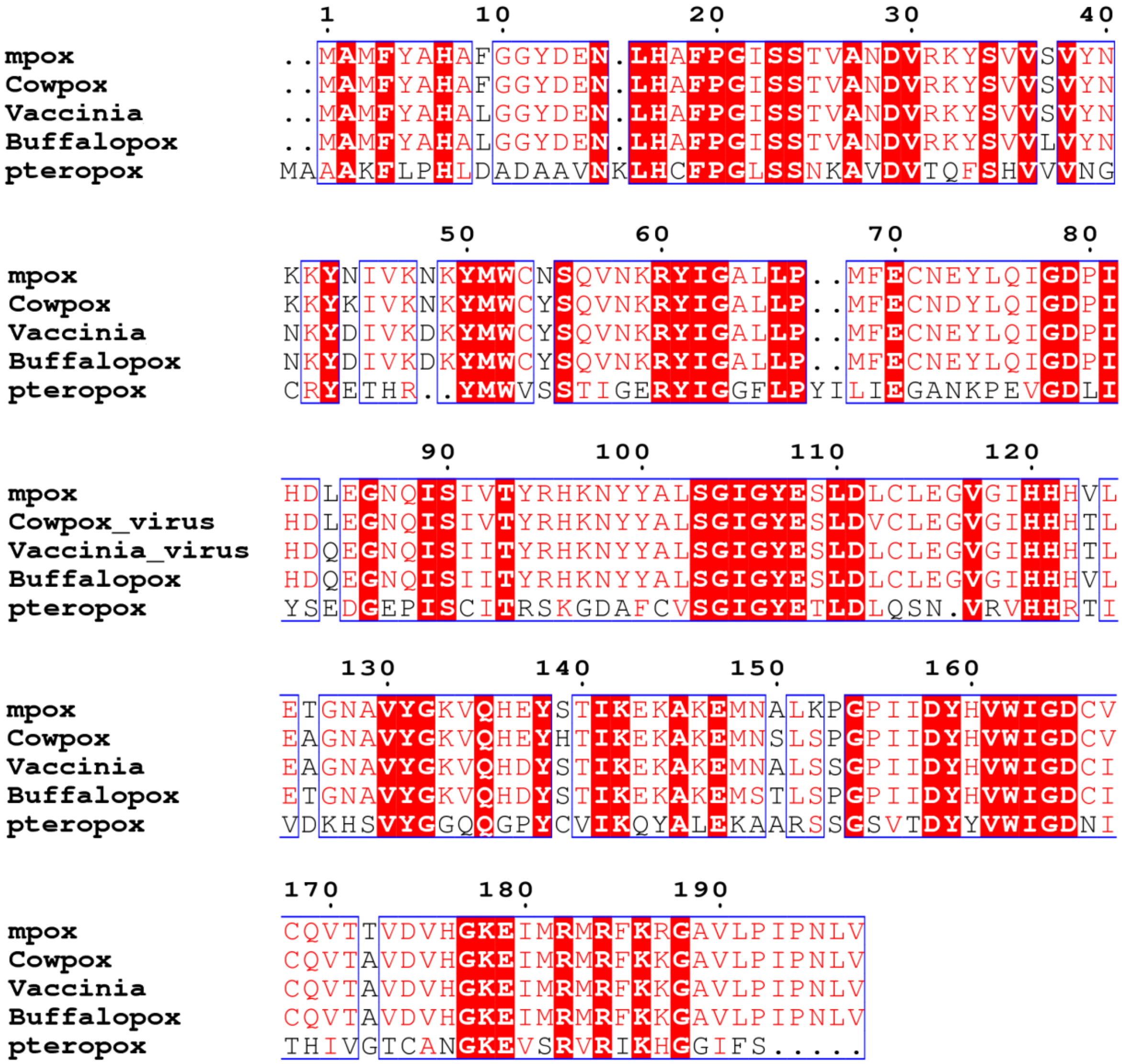

SI Figure 1: **Primary sequence alignment of the crystallized mpox poxin sequence with selected poxins.** Conserved residues are highlighted in red. The alignment was made in ESPript 3.0 server (https://espript.ibcp.fr/ESPript/ESPript/).

Materials and methods

Protein expression and purification - The gene encoding the mpox poxin (variant MPXV-W_Nigeria-156, GenBank: AIE40776.1, AAs 1-197) was commercially synthesized (Thermo Fisher Scientific) and cloned into a pSUMO vector which added a sequence encoding an 8xHis-SUMO purification and solubility tag. The protein was purified using our usual protocols for viral enzymes [1, 2]. Briefly, the expression plasmid was transformed into *E. coli* BL21 Star bacterial cells and cultured in LB medium supplemented with 100 µg/mL of ampicillin. The culture was left to shake overnight at 37 °C and then used to inoculate ZY5052 autoinduction media. At an optical density of 1.0 (OD_{600}) at 37 °C, the temperature was lowered to 18 °C and the culture was left to grow overnight. Collected cells were disrupted by sonication in lysis buffer (50 mM Tris pH 8.0, 300 mM NaCl, 20 mM imidazole, 10% glycerol and 3 mM β-mercaptoethanol). The 8xHis-SUMO-poxin fusion protein was purified from clarified lysates at 4 °C using Ni-NTA resin (Machery-Nagel) by the batch technique. Ni-NTA resin was extensively washed with lysis buffer adjusted to 1 M NaCl concentration. The protein was eluted by the elution buffer (50 mM Tris pH 8.0, 300 mM NaCl, 300 mM imidazole, 10% glycerol and 3 mM β-mercaptoethanol) and cleaved by the SUMO protease (recombinant Ulp1 protease) while being dialyzed against the lysis buffer. The 8xHis-SUMO tag was removed by an inverse Ni-NTA chromatography. The recombinant poxin was further purified using the size-exclusion chromatography on a Superdex 75 HiLoad 16/600 column (GE Healthcare) in a gel filtration buffer (20 mM HEPES pH 7.5, 250 mM KCl, 0.5 mM TCEP). The protein was concentrated to 15 mg/ml and stored in -80 °C until needed.

Protein crystallization - Initial crystals were obtained in the JCSG screens (QIAGEN), however, they were not of diffraction quality. They were used to prepare micro-seeds; a whole drop containing these crystals was collected, resuspended in100 µl seed buffer (100 mM HEPES pH 7.5, 20% w/v PEG 8000), intensively vortexed for five minutes and diluted to 10^{-4} in seed buffer. These seeds were immediately used; 250 nl of mother liqueur (100 mM HEPES pH 7.5, 5-10% w/v PEG 8000), 50 nl of seeds and 300 nl of poxin were mixed using the Mosquito robot (SPT Labtech) which resulted in diffraction quality crystals. The crystals were cryo-protected in mother liqueur supplemented with 20% v/v glycerol and flash frozen in liquid nitrogen.

Model building and refinement - The data were collected from a single crystal at the BESSY II MX beamline 14.1 [3]. XDS was used to process the data [4]. Crystals belonged to the monoclinic $P2_1$ spacegroup and diffracted to 1.7Å. The structure was solved by molecular replacement in Phenix [5] using chain A form the structure of vaccinia virus poxin (pdb code 6EA6) and manually refined in Phenix and Coot [6] to good statistics (Table 1).

Supplementary References

1. Dubankova, A., J. Humpolickova, M. Klima, and E. Boura, *Negative charge and membrane-tethered viral 3B cooperate to recruit viral RNA dependent RNA polymerase 3D (pol).* Sci Rep, 2017. **7**(1): p. 17309.
2. Konkolova, E., K. Krejcova, L. Eyer, J. Hodek, M. Zgarbova, A. Fortova, M. Jirasek, F. Teply, P.E. Reyes-Gutierrez, D. Ruzek, J. Weber, and E. Boura, *A Helquat-like Compound as a Potent Inhibitor of Flaviviral and Coronaviral Polymerases.* Molecules, 2022. **27**(6).
3. Mueller, U., R. Forster, M. Hellmig, F.U. Huschmann, A. Kastner, P. Malecki, S. Puhringer, M. Rower, K. Sparta, M. Steffien, M. Uhlein, P. Wilk, and M.S. Weiss, *The macromolecular crystallography beamlines at BESSY II of the Helmholtz-Zentrum Berlin: Current status and perspectives.* European Physical Journal Plus, 2015. **130**(7).
4. Kabsch, W., *Xds.* Acta Crystallogr D Biol Crystallogr, 2010. **66**(Pt 2): p. 125-32.
5. Liebschner, D., P.V. Afonine, M.L. Baker, G. Bunkoczi, V.B. Chen, T.I. Croll, B. Hintze, L.W. Hung, S. Jain, A.J. McCoy, N.W. Moriarty, R.D. Oeffner, B.K. Poon, M.G. Prisant, R.J. Read, J.S. Richardson, D.C. Richardson, M.D. Sammito, O.V. Sobolev, D.H. Stockwell, T.C. Terwilliger, A.G. Urzhumtsev, L.L. Videau, C.J. Williams, and P.D. Adams, *Macromolecular structure determination using X-rays, neutrons and electrons: recent developments in Phenix.* Acta Crystallographica Section D-Structural Biology, 2019. **75**: p. 861-877.
6. Emsley, P., B. Lohkamp, W.G. Scott, and K. Cowtan, *Features and development of Coot.* Acta Crystallogr D Biol Crystallogr, 2010. **66**(Pt 4): p. 486-501.
